# Association of the apparent diffusion coefficient with maturity in adolescent sacroiliac joints

**DOI:** 10.1002/jmri.25209

**Published:** 2016-02-21

**Authors:** Timothy J.P. Bray, Kanimozhi Vendhan, James Roberts, David Atkinson, Shonit Punwani, Debajit Sen, Yiannis Ioannou, Margaret A. Hall‐Craggs

**Affiliations:** ^1^ UCL Centre for Medical Imaging (Academic Radiology)LondonUK; ^2^ Arthritis Research UK Centre for Adolescent RheumatologyUniversity College LondonLondonUK

**Keywords:** diffusion‐weighted imaging, apparent diffusion coefficient, adolescents, inflammation, arthritis

## Abstract

**Purpose:**

To determine the extent to which apparent diffusion coefficient (ADC) values vary with skeletal maturity in adolescent joints.

**Materials and Methods:**

A retrospective study was performed with Institutional Review Board (IRB) approval. We used a picture archiving and communication system (PACS) search to identify and recruit all adolescents who had undergone 1.5T magnetic resonance imaging (MRI) of the sacroiliac joints (SIJs) between January 2010 and June 2015, and had no evidence of sacroiliitis and normal inflammatory markers. In all, 55 individuals were assessed. For each patient, coronal and sagittal images of the sacrum were visually analyzed to determine sacral maturity. Patients were divided into three groups depending on the degree of fusion of the sacral segmental apophyses: “Fused,” “Partial,” and “Unfused.” For each group, SIJ ADC was measured using a linear region‐of‐interest technique.

**Results:**

Mean ADC values were 690 × 10^−6^ mm^2^/s in the fused group, 720 × 10^−6^ mm^2^/s in the partial group, and 842 × 10^−6^ mm^2^/s in the unfused group. ADC values were significantly higher in the unfused group than in the fused group (*P* = 0.046). ADC values were also higher in unfused subjects than partially fused subjects (*P* = 0.074).

**Conclusion:**

Joint ADC values are higher in skeletally immature (unfused) patients than in skeletally more mature (fused) patients. ADC values measured in the unfused group overlap with those previously reported in sacroiliitis. These results suggest that ADC measurements in adolescent joints must be interpreted in light of joint maturity. Joint immaturity may lead to misdiagnosis of sacroiliitis, since immature juxta‐articular bone may appear similar to inflammation. J. Magn. Reson. Imaging 2016. J. Magn. Reson. Imaging 2016;44:556–564.

Enthesitis‐related arthropathy (ERA) is severe subtype of juvenile idiopathic arthritis (JIA), and carries a substantial burden of morbidity and disability.^1^ Sacroiliitis is a particular problem in ERA, and up to 80% of patients with low back pain in ERA have sacroiliitis.^2^ However, monitoring the disease is difficult because clinical symptoms may be nonspecific; clinical assessment has been shown to be an insensitive tool for assessing sacroiliitis as diagnosed using magnetic resonance imaging (MRI).^3^ An accurate, specific method for diagnosis and quantification of sacroiliitis is therefore an important part of these patients' management.

MRI has emerged as a valuable tool for assessing the sacroiliac joints.^4^ Conventional qualitative techniques rely on visual analysis of short tau inversion recovery (STIR) images,^5^ but recent research has demonstrated that diffusion‐weighted imaging (DWI) can be used as a fast, objective alternative.^6^, ^7^, ^8^, ^9^ However, assessment of joint inflammation in adolescence is challenging because the structure and composition of the sacroiliac joint changes substantially during skeletal maturation.

Sacral ossification begins in the first two sacral segments in utero.^10^, ^11^ As the sacral apophyses ossify during childhood, residual cartilaginous connections that link the SIJs and the neural foramina gradually disappear. The apophyses typically fuse between the ages of 16 and 20, although sometimes the SIJs remain immature well into late adolescence.^10^, ^12^ Immature sacroiliac joint morphology may be misinterpreted as inflammation because unossified cartilage causes juxta‐articular areas of high signal on STIR images (Fig. 1). To our knowledge, there are no previous studies investigating the influence of joint maturity on apparent diffusion coefficient (ADC) values as measured using DWI.

**Figure 1:**
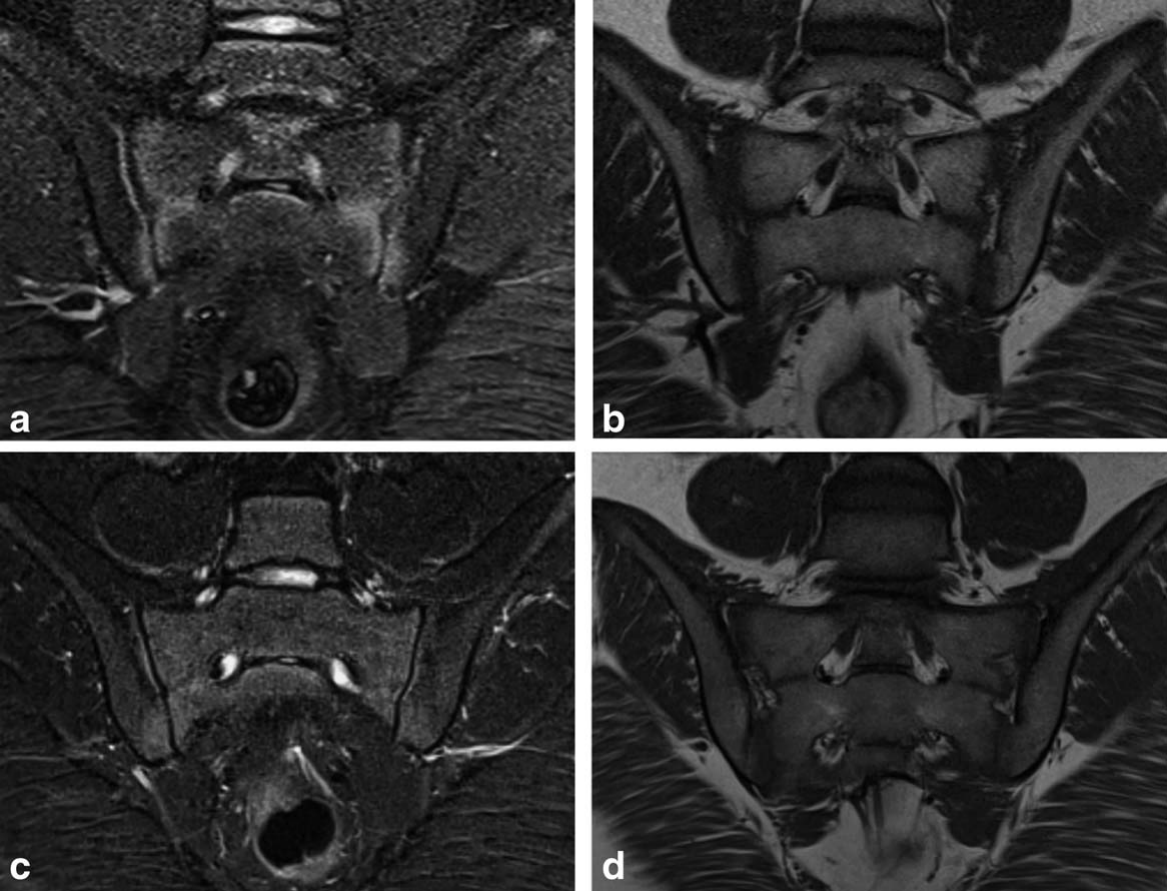
STIR and *T*_1_‐weighted images from an 11‐year‐old female **(a,b)**. In this case, the juxta‐articular bands of high signal on the STIR images were misdiagnosed as inflammation. Further scans **(c,d)** 2 years later (when the patient was 13 years old) show that the joint is maturing normally and the juxta‐articular bands of high signal are gradually disappearing. Clinically, the diagnosis was of mechanical back pain, and biochemical inflammatory markers were normal throughout.

We hypothesized that ADC values would vary with joint maturity, since unossified bone would be expected to contain a higher proportion of water than fully mineralized bone (the relationship between water content and ADC is well established^13^. Furthermore, we hypothesized that normalized ADC (nADC) values^14^ would also be higher in unfused SIJs. nADC values (ie, the mean SIJ ADC value divided by a “reference” ADC obtained from normal sacral bone) are of particular interest because nADC has been investigated as a biomarker of inflammation in sacroiliitis,^6^ and may be less susceptible to between‐scan variability than uncorrected ADC.

In this study we aimed to evaluate the association between SIJ maturity and both ADC and nADC measurements in adolescent and young adult patients with noninflammatory back pain.

## Materials and Methods

This study was covered by Institutional Review Board (IRB) approval (REC ref: 11/LO/0330) and informed consent was waived due to its retrospective nature.

### Subjects

**Figure 2:**
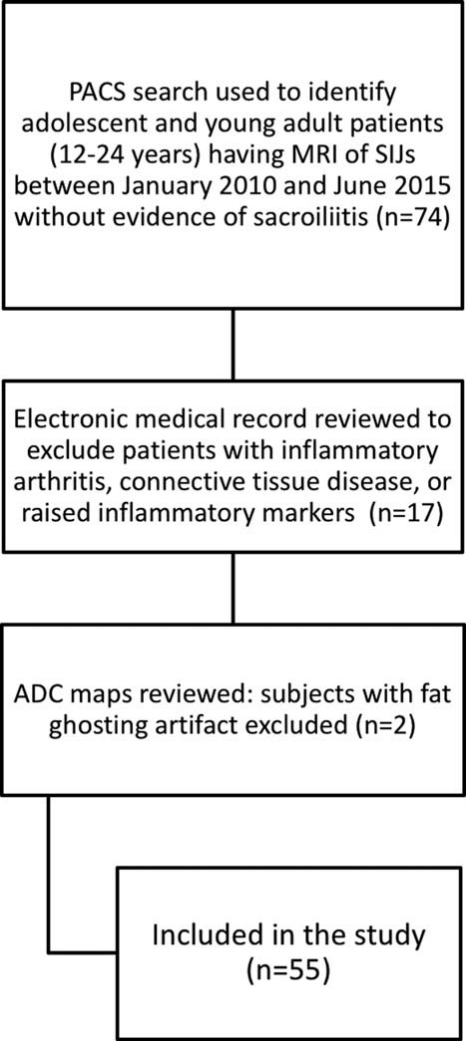
Flowchart demonstrating the process for study inclusions and exclusions.

A picture archiving and communication system (PACS) search was used to identify 74 adolescent and young adult patients (aged 12–24 years) who had an MRI of the sacroiliac joints performed at our institution from January 2010 to June 2015 without evidence of sacroiliitis. For all potential subjects, the electronic medical record was reviewed to ensure a final clinical diagnosis of mechanical, noninflammatory back pain and normal inflammatory markers (defined as a serum C‐reactive protein level less than 5 mg/L, and an erythrocyte sedimentation rate less than 7 mm/hr). Individuals with inflammatory arthritis or connective tissue disease were excluded (*n* = 17). Patients whose images were substantially degraded by artifact (particularly fat ghosting due to inadequate fat suppression) were also excluded (*n* = 2). A flowchart demonstrating the process for inclusion or exclusion from the study is shown in Fig. 2.

### MRI Technique

Images were acquired on a single 1.5T scanner (Avanto; Siemens, Erlangen, Germany), as shown in Table 1.

**Table 1:** MRI Acquisition Parameters

Sequence	Plane	Parameters
T1 turbo spin echo (TSE) coronal	Coronal	TR/TE 610/11ms, slices 18, slice thickness 3mm, FOV 200mm; T1 TSE axial – TR/TE 610/11ms, slices 18, slice thickness 3mm, FOV 200mm, matrix size 256 × 256, pixel size 1mm.
T1 TSE axial	Axial	TR/TE 475/11ms, slices 20, slice thickness 5mm, FOV 200mm; T1 TSE axial – TR/TE 610/11ms, slices 18, slice thickness 3mm, FOV 200mm, matrix size 256 × 256, pixel size 1mm.
Short tau inversion recovery (STIR)	Axial	TR/TE 6070/83ms, inversion time 150ms, slices 18, slice thickness 5mm, FOV 200mm, matrix size 256 × 256, pixel size 1mm.
T1 Turbo Inversion Recovery Magnitude	Coronal	TR/TE 4340/83ms, inversion time 150ms, slices 14, slice thickness 4mm, FOV 200mm, matrix size 256 × 256, pixel size 1mm.
Postcontrast T1 TSE with fat saturation	Axial	TR/TE 619/11ms, slices 20, slice thickness 5mm, FOV 200mm, matrix size 256 × 256, pixel size 1mm.
Postcontrast T1 TSE with fat saturation	Coronal	T1 TSE fat sat coronal ‐ TR/TE 795/11ms, slices 18, slice thickness 3mm, FOV 200mm, matrix size 256 × 256, pixel size 1mm.
Diffusion‐weighted images	Axial	Single‐shot DWI with EPI readout. TR/TE 3500/87, FOV 316mm, matrix size 128 × 128, pixel size 2.5mm, slice thickness 8mm, averages 4, slices 17, EPI factor 120, b‐values 0, 50, 100, 300 and 600s/mm^2^ with fat saturation. ADC maps were generated on vendor software using a standard monoexponential fit. GRAPPA was used to reduce distortion (acceleration factor 2).

TR, repetition time; TE, echo time; FOV, field of view; TSE; turbo spin echo; EPI, echo planar imaging; ADC, apparent diffusion coefficient; GRAPPA, generalized autocalibrating partially parallel acquisitions.

### Classification According to Maturity

Axial and coronal *T*_1_‐weighted images and coronal STIR were reviewed in consensus by two observers (M.H.C. and T.B., with over 20 years and 4 years of musculoskeletal MR experience, respectively) to determine the degree of maturity of the SIJs. The joints were assessed at S1/2 and S2/3. Based on previous work describing age‐related differences in the degree of fusion of the segmental apophyses,^12^ subjects were classified as either “unfused,” “partially fused,” or “fused” as follows.

#### Unfused

**Figure 3:**
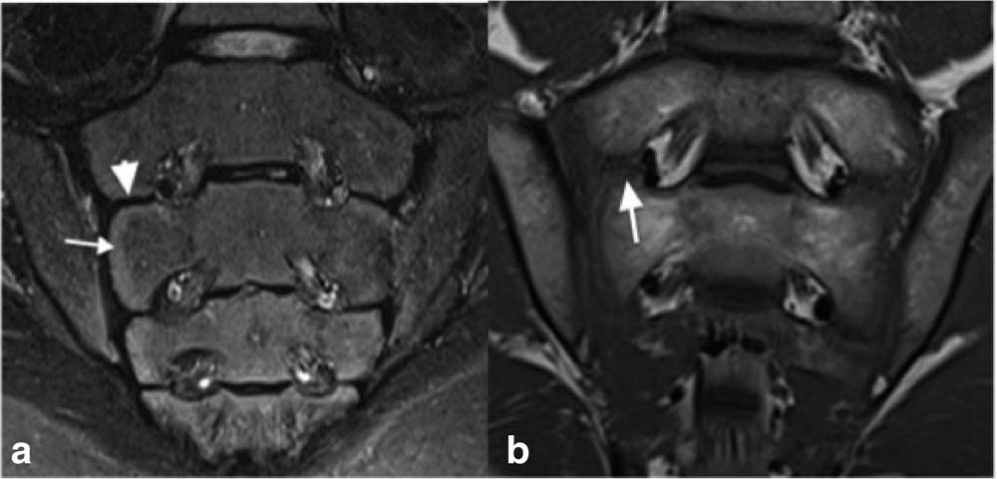
Coronal STIR **(a)** and *T*_1_W **(b)** images in a 14‐year‐old with immature SIJs, which demonstrates unfused intersegmental apophyses. The STIR image demonstrates the unfused persistent cartilaginous connection between the joint and neural foramina (arrowhead). There is a high signal band adjacent to the SIJ corresponding to immature, unossified bone (arrow). The *T*_1_W image also shows unfused intersegmental apophyses (arrow).

The apophyses between the sacral segments are unfused (open), and there is a complete cartilaginous connection between the SIJs and neural foramina (Fig. 3). There are no areas of bony fusion visible on either the coronal or sagittal images. On the STIR images, these patients often show high signal bands of unossified bone adjacent to the SIJ on the sacral side, in continuity with the unossified intersegmental bone, although this was not used as a classification criterion (Fig. 3).

#### Partially Fused

**Figure 4:**
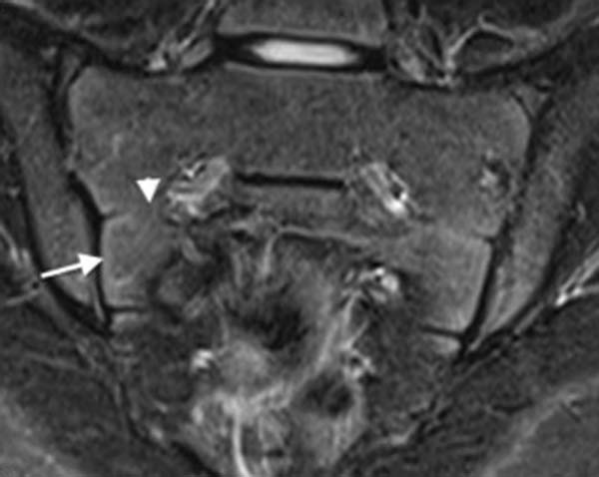
Coronal STIR image of an adolescent with partially fused intersegmental apophyses. Partial ossification (arrowhead) of the segmental apophyses between S1 and S2 is seen medially. In this patient, there remains a thin high signal band (arrow) of immature bone adjacent to the SIJ, but this is thinner than in a patient with completely unfused apophyses.

There are some areas of fusion (ie, there is an area of continuous bone joining the sacral segments) but the fusion is incomplete (Fig. 4). This group includes a spectrum of patients ranging from those with very early fusion, to those where fusion was almost complete (ie, there are small areas of residual unossified cartilage).

#### Fused

**Figure 5:**
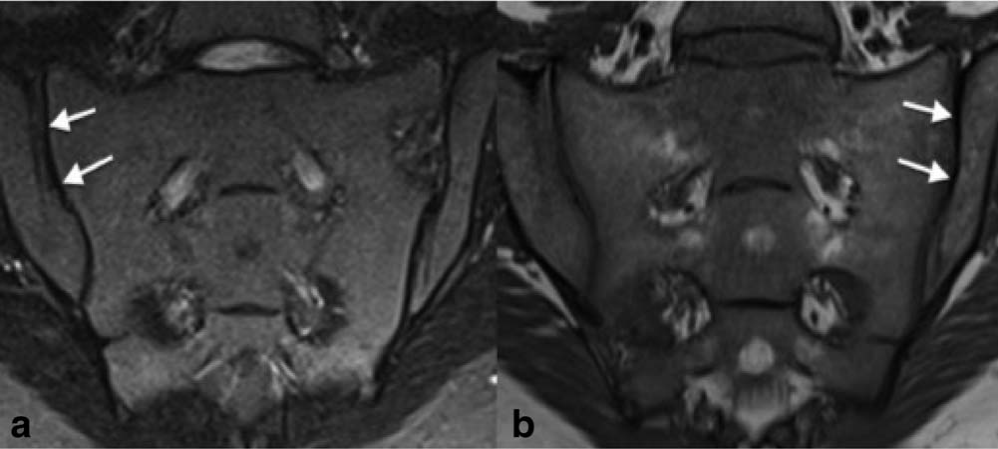
Coronal *T*_1_‐weighted image of an adolescent with fully fused intersegmental apophyses. The joint margin is clearly defined (arrow) and the cartilaginous bands linking the neural foramina and the SIJs have disappeared.

The intersegmental apophyses are fully fused. There is no residual unossified cartilage. The contours of the sacroiliac joint are sharply defined (Fig. 5).

### ADC Measurement

The ADC maps were analyzed with MatLab software (MathWorks, Natick, MA) using in‐house code and a previously developed quantification technique,^6^ as follows.

**Figure 6:**
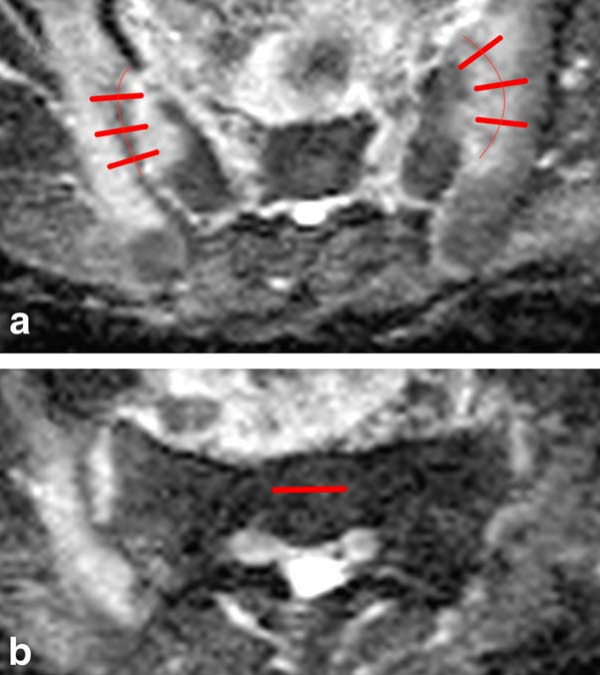
Placement of ROIs on ADC maps. **a**: Three linear ROIs are placed on both sacroiliac joints (thick red lines). The joint itself is shown as a thin red line. **b**: A further ROI is placed on interforaminal sacral bone, as previously described.^6^

Three linear regions of interest (ROIs) measuring 14 mm were drawn across the synovial portion of the SIJ (Fig. 6); where the anteroposterior dimensions of the joint were insufficient to accommodate three ROIs, only two were drawn. Each ROI was centered on the joint space. ROIs were placed on both joints on the four axial ADC slices that best represented the central portion of the SIJ (each with a slice thickness of 8 mm). The mean joint ADC was calculated for each patient. ADC measurements were performed by two observers: radiology residents (T.B. and J.R.) with 4 and 2 years of musculoskeletal MR experience, respectively, who were each blinded to the other observer's ROI placement and ADC measurements. The mean of the two individuals' mean joint values was taken as the “uncorrected” ADC value.

Additionally, a further “reference” ROI was placed on normal sacral bone by each observer to enable calculation of normalized ADC values. The normalized ADC (nADC) value of each patient was defined as the ratio between the mean ADC of all joint line profiles and the mean ADC of the reference ROI from normal sacral bone.

### Statistical Methods

Mean ADC, nADC, and reference ADC values for each subject were compared between the three groups using balanced one‐way analysis of variance (ANOVA). A post‐hoc multiple comparison test (Tukey's honestly significant difference criterion) was used to establish specific differences between groups. Additionally, we used a multilevel mixed‐effects linear regression analysis to compare ADC values from *individual* ROIs between the three groups, using observers and subjects as grouping variables. The “Unfused” group was used as the baseline for comparison. The ages of the three groups were compared using a one‐way ANOVA, and sex was compared between the three groups using a 2×3 Fisher's exact test.

Interobserver variability was assessed for ADC, nADC, and reference ADC values using Bland–Altman 95% limits of agreement and intraclass correlation coefficient (absolute agreement).

## Results

### Demographics

Fifty‐five subjects were included in the study, with a mean age of 15 years 11 months (range 10 years 2 months to 18 years 11 months).

Demographics for the three groups are summarized in Table 1. Patients in the unfused group were significantly younger than those in the fused group (*P* = 0.011) and also younger than those in the partial group (*P* = 0.051). There was no significant difference in the ages of subjects in the partial and fused groups (*P* = 0.53).

There were 36 females and 19 males in the group as a whole. Of these, six females were classified as unfused, 22 as partially fused, and eight as fused. Six males were classified as unfused, seven as partially fused, and six as fused. There was no significant association between sex and fusion class (*P* = 0.22, Fisher's exact test).

### Comparison of Fused, Partially Fused, and Unfused Groups

ADC, nADC, and reference ADC values for the three groups (fused, partially fused, and unfused) are shown in Table 2.

**Table 2:** Demographic Information for Fused, Partially Fused, and Unfused Groups

	Fused	Partially fused	Unfused
Subjects	14	29	12
Males (%)	6 (43%)	7 (24%)	6 (50%)
Mean age (SD)	16y 7m (1y 6m)	16y 1m (1y 4m)	14y 10m (1y 11m)
Age range	5y 4m (13y 8m to 18y 11m)	5y 7m (12y 7m to 18y 2m)	6y 10m (10y 2m to 16y 11m)

**Table 3:** Mean (Standard Deviation) ADC and nADC Values for Fused, Partially Fused, and Unfused Groups

	Fused	Partially fused	Unfused
ADC (mm^2^/s × 10^−6^)	690 (174)	720 (156)	842 (145)
nADC	1.23 (0.14)	1.34 (0.35)	1.40 (0.27)
Reference ADC (mm^2^/s × 10^−6^)	571 (140)	577 (132)	592 (131)

#### ADC

**Figure 7:**
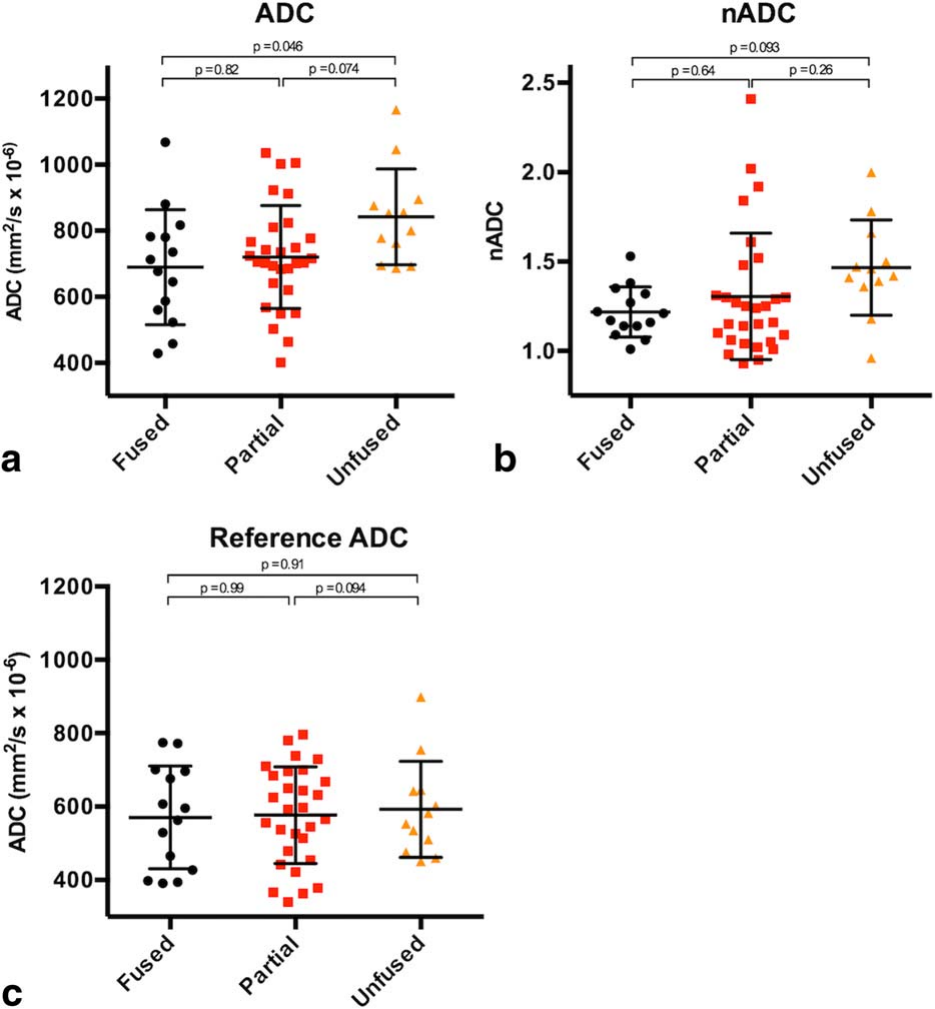
Boxplots showing the comparison of fused, partial, and unfused SIJ groups according to uncorrected ADC values **(a)**, normalized ADC values **(b)**, and reference ADC values **(c)**. The central lines for each group represent the mean; the bars represent the standard deviation.

The mean ADC values were 690 ± 174 × 10^−6^ mm^2^/s in the fused group, 720 ± 156 × 10^−6^ mm^2^/s in the partial group, and 842 ± 145 × 10^−6^ mm^2^/s in the unfused group (Fig. 7a). Mean ADC values were significantly higher in the unfused group than in the fused group (*P* = 0.046). Mean ADC values were also lower in the unfused group than in the partially fused group (*P* = 0.074) but there was no significant difference between partially fused and fused groups (*P* = 0.82). Accordingly, multilevel regression analysis found that individual ADC values in the unfused group were significantly higher than those in the fused group (*P* = 0.019, regression coefficient: –133 × 10^−6^ mm^2^/s, 95% confidence interval [CI]: –233 to –34) or the partial group (*P* = 0.009, regression coefficient: –124 × 10^−6^ mm^2^/s, 95% CI –227 to –20).

#### nADC

The mean nADC values were 1.23 ± 0.14 in the fused group, 1.34 ± 0.35 in the partial group, and 1.40 ± 0.27 in the unfused group (Fig. 7b).

The difference between fused and unfused groups showed a trend towards statistical significance (*P* = 0.093). There was no significant difference between partial and fused groups (*P* = 0.64) or between partial and unfused groups (*P* = 0.26).

#### Reference ADC

Reference ADC values were 571 ± 140 × 10^−6^ mm^2^/s in the fused group, 577 ± 132 × 10^−6^ mm^2^/s in the partial group, and 592 ± 131 × 10^−6^ mm^2^/s in the unfused group (Fig. 7c). There was no significant difference in reference ADC between any of the three groups (fused compared to partial: *P* = 0.99, fused compared to unfused: *P* = 0.91, partial compared to unfused: *P* = 0.94).

### Reproducibility

**Figure 8:**
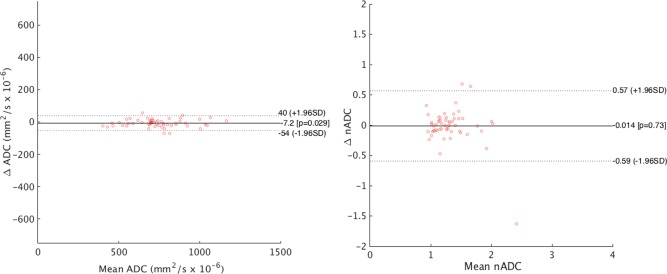
Bland–Altman plots demonstrating interobserver variability for mean ADC (left) and mean normalized ADC (right). Each data point represents one subject (*n* = 55).

Bland–Altman plots for ADC and nADC reproducibility are shown in Fig. 8.

For the ADC analysis, the Bland–Altman 95% limits of agreement were ±47 across a range of values from 401 to 1166. The intraclass correlation coefficient was 0.99. The coefficient of variance was 0.23.

For the nADC analysis, the Bland–Altman 95% limits of agreement were ±0.58 across a range of values from 0.93 to 2.41. The intraclass correlation coefficient was 0.62. The coefficient of variance was 0.23.

For the reference ADC, the Bland–Altman 95% limits of agreement were ±160 across a range of values from 340 to 579. The intraclass correlation coefficient was 0.84. The coefficient of variance was 0.22.

## Discussion

Recent work has examined the use of DWI as a tool for quantifying sacroiliitis in adolescents and young adults with ERA.^6^ However, the sacroiliac joints undergo substantial structural changes during adolescence^10^, ^12^ and SIJ ADC values might therefore be expected to vary according to skeletal maturity. In this study we found that SIJ ADC values were higher in patients with unfused sacral segmental apophyses compared to those with fused segmental apophyses. To our knowledge, this is the first report describing an association between skeletal maturity and SIJ ADC measurements.

ADC values in unfused subjects might be higher than in fused and partially fused subjects because the proportion of unmineralized bone adjacent to the joint is higher. Immature bone consists of cartilage and unossified or partially ossified bone, and would therefore be expected to contain a higher proportion of water than fully mineralized bone.^15^ ADC measurements have been shown to correlate with water content and collagen matrix structure in cartilage^16^, ^17^, ^18^ and variations in water content are related to the degree of mineralization of the bone matrix.^19^ Age‐ and subject‐related variations in the composition of bone marrow adjacent to the joint may also influence the measured ADC values, since red marrow displays significantly higher apparent diffusivity than yellow marrow.^20^, ^21^

Importantly, our results suggest that the joint ADC values in unfused subjects may overlap with those previously reported in sacroiliitis.^6^, ^7^, ^8^, ^9^ For example, Vendhan et al^22^ report a mean joint ADC value of 1211 × 10^−6^ mm^2^s^−1^ in ERA cases with sacroiliitis, which is only slightly higher than the upper end of the “unfused” normal range reported in this study (range 687 to 1166 × 10^−6^ mm^2^s^−1^). In adults, reported ADC values in sacroiliitis vary widely from 480 × 10^−6^ mm^2^s^−1^,^8^ to 1310 × 10^−6^ mm^2^s^−1^,^9^ while focal areas of bone marrow edema in adolescents with chronic nonbacterial osteomyelitis have been measured at 1600 × 10^−6^ mm^2^s^−1^.^23^ These results provide a clear indication that ADC values in adolescent joints cannot be viewed in isolation and must be interpreted in the light of joint maturity.

This work provides new information regarding the reproducibility of ADC values in normal adolescent SIJs. In our study the reproducibility of ADC values was excellent. Interestingly, the use of a reference ADC to normalize the data reduced the intraclass correlation coefficient and widened the Bland–Altman limits of agreement. This suggests that the use of uncorrected ADC values may provide better interobserver reproducibility than nADC, and may explain why the difference between fused and unfused groups was significant for ADC measurements and nonsignificant for nADC measurements. However, the use of a reference ADC may help to minimize scan variability that may occur due to the use of different scanning platforms when serial scans are acquired during patient treatment. We have been unable to assess this in the current study, as these patients had a single scan only.

A limitation of the current study is that joint maturation is a continuous process, and classification of borderline patients into discrete groups can be difficult. Nonetheless, there is a very clear distinction between the fused and unfused groups we describe. The group of patients with partial fusion of the segmental apophyses is more heterogeneous, and includes patients whose apophyses have only just started to fuse or are almost fully fused. Additionally, these patients were only scanned at a single timepoint—it was not possible to observe a progression in ADC changes over time in individual patients. In this study we only assessed apophyseal fusion at S1/2 and S2/3 since the apophyses below these levels are smaller—as a result, we could not consistently distinguish between partially fused and fused/unfused apophyses at S3/4 or S4/5 due to partial volume effects.

Another limitation of the present study is its retrospective nature. Ideally, one could prospectively recruit equal numbers of patients for the three groups (fused, partial, and unfused), although this would be rather impractical because an MRI scan is required for classification purposes. Furthermore, the scans were only acquired on a single scanner at one institution. It would be desirable to repeat this study prospectively in a larger cohort, ideally using multiple imaging platforms.

Further work will be required to develop strategies to allow for joint immaturity when quantitatively measuring inflammation of the sacroiliac joints. One approach would be to define a normal range of ADC values at different stages in sacral maturation. In JIA, it may be more practical to simply monitor joint ADC over time in individual patients since maturation would be expected to produce a gradual decrease in ADC, whereas inflammation causes joint ADC to increase.

In conclusion, we have shown that SIJ ADC values are higher in skeletally immature (unfused) patients than in skeletally more mature (fused) patients. ADC values measured in the unfused group overlap with those previously reported in sacroiliitis, suggesting that ADC measurements in adolescent joints must be interpreted in the light of joint maturity. Joint immaturity may lead to misdiagnosis of sacroiliitis, since immature juxta‐articular bone may appear similar to inflammation.
